# Tumour-Associated Macrophages (TAMs) in Colon Cancer and How to Reeducate Them

**DOI:** 10.1155/2019/2368249

**Published:** 2019-02-25

**Authors:** M. A. F. Yahaya, M. A. M. Lila, S. Ismail, M. Zainol, N. A. R. Nik Mohd Afizan

**Affiliations:** ^1^Faculty of Biotechnology & Biomolecular Science, Universiti Putra Malaysia, Serdang, Selangor, Malaysia; ^2^Faculty of Veterinary Medicine, Universiti Putra Malaysia, Serdang, Selangor, Malaysia; ^3^Herbal Medicine Research Centre, Institute for Medical Research, Jalan Pahang, Kuala Lumpur, Malaysia; ^4^Institute of Tropical Forestry and Forestry Products, Universiti Putra Malaysia, 43400 Serdang, Selangor, Malaysia

## Abstract

Tumour-associated macrophage (TAM) serves as the site in which most inflammatory cells coreside. It plays an important role in determining the progression and metastasis of a tumour. The characteristic of TAM is largely dependent on the stimuli present in its tumour microenvironment (TME). Under this environment, however, M2 macrophages are found to be in abundance compared to M1 macrophages which later promote tumour progression. Numerous studies have elucidated the relationship between TAM and the progression of tumour; hence, TAM has now been the subject of interest among researchers for anticancer therapy. This review discusses the role of TAM in colorectal cancer (CRC) and some of the potential candidates that could reeducate TAM to fight against CRC. It is with hope that this review will serve as the foundation in understanding TAM in CRC and helping other researchers to select the most suitable candidate to reeducate TAM that could assist in enhancing the tumouricidal activity of M1 macrophage and eventually repress the development of CRC.

## 1. Introduction

Colorectal cancer (CRC), also known as bowel cancer or colon cancer, is a type of cancer that begins its abnormal cell growth in the colon or rectum part of the large intestine. In men, CRC has been reported to be the third most common case to occur. On the other hand, it is the second most commonly occurring cancer incidence among women in the world [1, 2]. According to Ferlay et al. [3], approximately 1.36 million people around the world have been affected with cancer.

As shown in Table 1, in the United States of America (USA) alone, it is estimated that about 71,420 and 64,010 new CRC cases will be reported among males and females, respectively [4]. Meanwhile, in Asian countries, the disease has become one of the major health concerns in which the number of CRC cases being reported is increasing in an alarming state [5]. Among Asian countries, Japan has been recorded to have the highest incidence of CRC, followed by Hong Kong [6, 7]. Malaysia, in particular, has categorised CRC to be its second most common cancer reported.  Table 2 shows that ethnic Chinese holds the highest incidence rate per 100,000 (27.35), followed by Malay (18.95), and Indian (17.55) [8].

**Figure 1:**
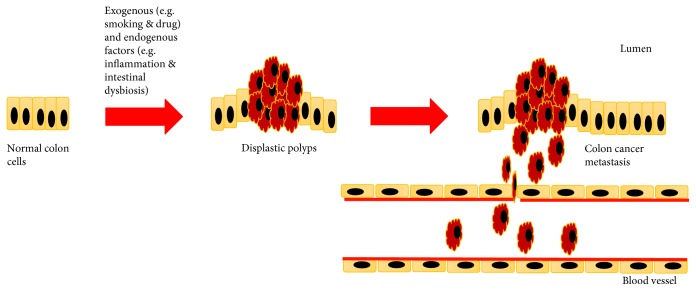
The metastasis of CRC in the presence of exogenous and/or endogenous factors (adapted and modified from Carini et al. [92]).

Several risk factors have been identified to be associated with the development of CRC. Obesity, lack of physical activity, tobacco use, moderate-to-heavy alcohol consumption, hypertension, abnormal blood lipids, and colonisation of *Streptococcus gallolyticus* (*S. gallolyticus*) are said to be the exogenous risk factors. Conversely, personal or familial history of colorectal polyps, inherited CRC syndromes (e.g., hereditary nonpolyposis colon cancer (HNPCC) and Cowden's disease), inflammatory bowel disease, and type 2 diabetes are said to be the endogenous risk factors [9–11]. As shown in Figure 1, in the presence of exogenous and/or endogenous risk factors, cancer development might occur if the risk factors managed to interact synergistically [12].

A comprehensive study conducted by Kannen et al. [13] showed that a slight increase of fat content in rat's daily diet may promote colonic preneoplasia. The promotion of colon carcinogenesis is due to the formation and continuation of colonocyte mutation. In addition, the reactivity between the established colon preneoplastic lesions with the tumour microenvironment (TME) depends on the amount of visceral adipose tissue present. This claim can also be supported by similar research done by Frajacomo et al. [14], in which the occurrence of DNA damage will orchestrate the changes of tissues in its microenvironment to promote colonic preneoplasia.

The microenvironment of colonic preneoplasia is an environment which the tumour might be originated and has a vital role in determining tumour initiation, progression, or regression [15]. Once the tumour has established, its microenvironment can now be referred to as TME. According to Wang et al. [16] and Chen et al. [17], TME consists of extracellular matrix (ECM), myofibroblasts, and a number of cellular players (e.g., neuroendocrine cells, immune-inflammatory cells, and lymphatic vascular networks). TME is a unique environment that develops in the sequence of tumour progression as a result of its interactions with the host. The current review provides an understanding on the role of TAM in colorectal cancer (CRC) and some of the potential candidates that could reeducate TAM to fight against CRC.

## 2. Tumour-Associated Macrophage (TAM)

As described in Section 1, TME will be created when the tumour has established in an area that is dominated by tumour-induced interactions [18]. A number of studies have shown that most human tumours are infiltrated with inflammatory cells such as eosinophils, mast cells, neutrophils, and macrophages [19–21]. The presence of these infiltrated inflammatory cells together with their cytokines will orchestrate the molecular and cellular events at the neighbouring tumour tissue site. However, this review will only be focusing on the action of macrophages in regulating tumour development, concentrating on colon tumour.

Macrophages are, generally, said to be the most dominant leukocyte residents found in the TME compared to other components such as stromal cells and mast cells [22, 23]. Macrophages are part of the mononuclear phagocytic system (MPS) which plays a pivotal role in the host innate immune response against any pathogenic infections [24]. Furthermore, macrophages are also involved in tissue homeostasis, inflammation, and tissue repair and development [24, 25]. Conventionally, there are two types of macrophages, namely, M1-like macrophage (M1 macrophage) and M2-like macrophage (M2 macrophage). M1 macrophages perform an important role in the innate response against pathogenic infection, while M2 macrophages act in tissue repair and tumour progression. The ability of macrophages to repolarise from M1 macrophage into M2 macrophage, vice versa, gives a unique characteristic to the cell which reflects the indecisive relationship between TAM and the cancer cells [26]. This ability is referred to as plasticity of macrophages. The subversion of M1/M2 macrophages is due to the environmental cues present in the TME which will eventually affect the adaptive immunity and inflammatory circuits in promoting tumour growth and progression [27–29]. The mechanism as to how macrophages are differentiated will be further elaborated in the following section.

The macrophages found in the TME are often referred to as tumour-associated macrophages (TAMs) which are mostly in M2 phenotype form [26]. TAMs are frequently immune cells that play the major role in promoting the tumour cells in the TME by inhibiting T cell-mediated antitumour immune response [24, 30, 31]. At the primary site of a tumour, TAMs will either directly enhance the cell growth of the tumour by promoting angiogenesis or indirectly induce the dysfunction of immune cell interaction within the TME. TAMs can promote the development of tumour via different mechanisms, which will be further explained in details in the subsequent sections of this review.

## 3. Phenotypical Distinction of Macrophage Subtypes

Macrophages are characterised distinctively depending on the growth factors and chemokines present in its TME. For example, M1 macrophage will be polarised if there are any inducers (e.g., interferon-*γ* (IFN-*γ*), lipopolysaccharides, or tumour necrosis factor *α*) present in the TME [32]. On the other hand, in the presence of interleukin 4 (IL-4) and IL-13, the monocytes will be polarised into M2 macrophages. The inducers can either independently or coincite the monocyte to become M1 or M2 macrophage. The polarisation of M1 macrophage from monocyte is often referred to as classical activation, whereas alternative activation is frequently referred to the polarisation of M2 macrophage. The M1 and M2 macrophage polarisation is analogous to the polarisation of T-helper 1 (Th1) and Th2 cells [22, 33].

Phenotypically, M1 macrophage is characterised by its ability to express high level of proinflammatory cytokines, promotion of Th1 response, high production of reactive nitrogen and oxygen intermediates, and strong tumouricidal activity [34]. In contrast, M2 macrophage is characterised by its ability to promote tissue remodelling and tumour progression, high scavenging molecules, and effective phagocytic activity [35]. Under certain physiological and pathological conditions, M2 macrophage is able to repolarise into M1 macrophage, vice versa, due to its high-plasticity feature [36, 37]. However, the molecular mechanisms that regulate the phenotypical macrophage switching remains poorly understood.

**Figure 2:**
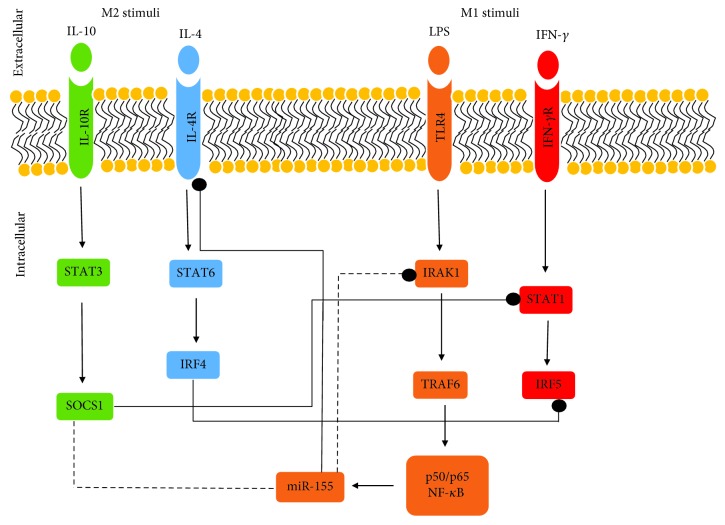
The polarisation of M1 and M2 macrophages from monocytes in the presence of their microenvironmental cues. The arrows indicate the effect from positive regulation (induction or activation), bullet-end lines indicate the effect from negative regulation (inhibition/inactivation), and the dotted lines indicate direct targets of the miRNA (adapted and modified from Mantovani and Locati [32]).

The polarisation of M1 and M2 macrophages by their respective inducers will intercellularly initiate the canonical signalling pathways (Figure 2). For instance, the attachment of IFN-*γ* onto its receptor (IFN*γ*-R) located on the cell surface of monocyte cell will initiate the activation of signal transducer and activator of transcription (STAT) 1 signalling pathway to give rise to M1 macrophage [38]. Upon M1 macrophage polarisation, Th1 cell responses will be upregulated. Th1 response possesses robust tumouricidal and microbicidal activities [39]. In addition, proinflammatory cytokines (e.g., TNF-*α*, IL-6, and IL-12) will be upregulated as well.

M2 macrophage, on the other hand, will be polarised upon induction by its stimuli e.g., IL-4, IL-13, and IL-10 [40, 41]. As shown in Figure 2, the polarisation of M2 macrophage begins when IL-4, for example, engaged with its receptor, IL-4R, on the surface of monocyte cell. The IL-4 and IL-4R engagement promotes the activation and translocation of STAT6 via Janus kinase 1 (JAK1) and JAK3 [42].

Interferon regulatory factor (IRF)/STAT signalling acts as the central pathway in regulating the polarisation of M1-M2 macrophage. However, in the case of some disease progression, nuclear factor-*κ*B (NF-*κ*B) acts as the key regulator of macrophage plasticity by spatiotemporal activation [39].

NF-*κ*B is normally being kept in its inactive form in CRC [43]. This can be seen in a study conducted by Greten et al. [44] in which the inactivation of NF-*κ*B significantly reduced the expression of certain genes (e.g., IL-1*β*, TNF-*α*, and macrophage inflammatory protein 2 (MIP-2)) involved in inflammatory response. On the contrary, the same study also showed that the functional abolition of NF-*κ*B in mice's intestinal epithelial cells did not influence the degree of inflammation. However, it resulted in significant reduction of tumour size due to the enhanced epithelial cell apoptosis during early tumourigenesis. From this study, it suggests that the activation of NF-*κ*B controls the survival of transformed cells and leukocyte-driven inflammation which eventually provides the signalling molecules that act in sustaining tumour growth.

## 4. TAM in Colon Tumour

Several studies have shown that TAM originated from monocytic predecessors that circulate in the blood streams which has protumoural functions [45–47]. In order for monocytes to be able to reach the tumour site, they require chemokines that act as the chemotactic factors to signal the monocytes to directly travel to the tumour site. Such chemokines are CCL2, vascular endothelial growth factor (VEGF), CCL5, and TGF-*β* [48–50]. The recruited monocytes at the tumour site will undergo differentiation to become mature macrophages in the TME (refer to Section 3).

Albeit several studies have shown that the increasing number of macrophages infiltrated in the TME correlates with the improved survival rate among CRC patients [51, 52], the studies conducted by Sickert et al. [53] and Nakayama et al. [54] reported that decreasing macrophage number is associated with a more advanced stage of CRC among CRC patients. In this incident, the decreased number of CD68^+^ macrophages was due to the presence of VEGF isoforms [55] responsible for promoting the angiogenesis of the tumour [56].

TAM in CRC was shown to be able to promote angiogenesis and metastasis due to its ability to secrete VEGF [57]. The secretion of VEGF is due to the activation of NF-*κ*B that has been induced by the IL-1, IL-6, and TNF-*α* cytokines produced by the TAM [58]. IL-6, for example, is responsible for promoting colon tumourigenesis by inducing the production of STAT3-mediated IL-10 in tumour cells [59]. According to Bollrath et al. [60], STAT3 acts to enhance the nuclear localisation of *β*-catenin which plays a role in cell-to-cell interaction in growth regulation.

Despite the numerous literatures found on TAM protumoural function and poor prognosis, some studies have shown that TAM in CRC demonstrates to have antitumour activity and is linked with enhanced disease-free survival [51, 61, 62]. Sugita et al.'s [63] work has proved that this concept is correct by demonstrating that the macrophages and tumour margin are able to induce cell death in Fas ligand-dependent manner. The relationship between cancer cell death and hematogenous metastasis is inversely proportional which translates the protective property of TAM in tumourigenesis [63]. In support of Sugita et al.'s [63] study, Forssell et al. [51] also demonstrated that a dense macrophage infiltration at the colon tumour site will give a positive influence towards colon cancer prognosis in CRC patients. However, direct macrophage-to-tumour cell contact is needed in order to manifest the antitumourigenic activity by TAM.

The antitumour and protumour activities by TAM depend on the hypoxic condition of its environment [43, 64]. In the context of tumourigenesis, hypoxic condition refers to the condition in which the tumour cells are experiencing oxygen deprivation resulting in rapid angiogenesis. The angiogenesis will form in an area with significant lower oxygen concentration among healthy tissues. In addition, the hypoxic condition promotes healthy cells to behave abnormally due to the remodelling of its extracellular matrix which eventually increases the metastatic and migratory progressions [65, 66].

The hypoxic environment does not only affect the healthy cells to become cancerous cells; it also affects the behaviour of TAM. For instance, TAM will be most likely to become tumouricidal if it is located in an area with less hypoxic condition and less exposure of tumour-derived cytokines [44]. Such circumstance will occur due to the presence of significant number of M1 macrophages [64]. In a situation in which tumour development has entered the advanced stage, most macrophages will have the tendency to polarise into M2 macrophages, thus having protumour function [67]. Therefore, it is of pivotal requirement for macrophages to be polarised into M1 macrophages instead of M2 macrophages to ensure that the colon tumour cells can be regressed. However, the number of M2 macrophages is rather higher than M1 macrophages in TME which resulted in the difficulty of the colon tumour cells to be regressed [68].

## 5. Potential Candidates to Reeducate TAM

TAM is prominently found in the TME of most malignant types in which the majority of them being M2 macrophages [69, 70]. The presence of abundant M2 macrophage number in the TME resulted in the initiation and continuous growth of tumour. Hence, researchers have shown their strong interest in designing a number of strategies to target TAM for cancer therapy [30, 71, 72]. Though there are few strategies to target TAM for cancer therapy as mentioned by Tang et al. [71], this review will be focusing on reeducating TAM by disrupting certain molecular pathways that contribute to the repolarisation of M1 to M2 and also promote the repolarisation of M2 to M1 as part of the enhancement strategy for M1 tumouricidal activity [73].

The plastic property of macrophage has sparked the idea of most scientists to make use of this property to reeducate TAM. As TAM consists of high number of M2 macrophages, there is a possibility to reeducate TAM by repolarising the protumoural M2 macrophages to become tumouricidal M1 macrophages. However, the mechanism to reeducate TAM requires full understanding as it is involved with the complex pathways as shown in Figure 2. In addition, the local cytokine profiles located in the TME also play an important role in determining the successful rate of macrophage repolarisation.

M2 macrophages harbouring TAM can be activated by STAT3 and STAT6 transcriptional factors which allow the M2 macrophages to promote cancer advancement [74]. As an example, a recent study was conducted by Park et al. [73] on the assessment of immunohistochemistry of tissue samples from patients suffering from stages 1 to 3 CRC disease to investigate the cytoplasmic expression of STAT3. The study shows that the inflammatory cell infiltrate was downregulated due to the increased number of STAT3 expression. The observation from the study is in parallel with the observation done by Yu et al. [75] in which they observed that the activation of STAT3 will induce the differentiation of naïve T-lymphocytes to become tumour-promoting lymphocytic-type cells in cancer mouse model. Therefore, by blocking the expression of STAT3, it is possible to reeducate TAM from tumour-promoting to tumour-inhibiting macrophages. Interestingly, FLLL32, a diketone analogue of curcumin, has been designed in human colorectal cancer cell line (i.e., SW480, HCT116) study [76]. FLLL32 was designed in a way to be able to bind at the SH2 binding site of STAT3 molecule. At the concentration of 10 *μ*M, FLLL32 was able to inhibit the phosphorylation of STAT3 at the residue Ser727. FLLL32 can be the potential candidate in reeducating TAM to fight against colon cancer.

Apart from the importance of STAT pathway as mentioned above, the NK-*κ*B pathway also plays a critical role in ensuring the optimal M1 tumouricidal activity and the expression of proinflammatory cytokines [38]. The attenuation of NK-*κ*B pathway leads to the M2 macrophage immunosuppressive activity [77]. Interestingly, several agents have been identified that have the ability to hinder the NK-*κ*B pathway. One of the agents is the anti-CD40 antibody. Chen and Ross [78] have shown an interesting finding in which CD40 is able to inhibit the cytotoxic function of macrophages due to the ability of its ligands in inhibiting the trans-activity of NK-*κ*B canonical pathway in both human and rodent monocytic cell model. Such mechanism is crucial in promoting the gene expression of CXCL12 and VEGF-C, thus establishing the tumouricidal activity of M1 macrophage.

Peroxisome proliferator-activated receptors (PPARs) and hypoxia-inducible factors (HIFs) are another potential candidates to reeducate TAM. PPAR-*γ* is a subset of PPARs that acts as the transcriptional inhibitor of NF-*κ*B which leads to the differentiation of M2 macrophage [79]. PPAR-*γ* blocks NF-*κ*B by antagonising the polarisation of M1 macrophage and supporting the M2 macrophage polarisation [80]. On the other hand, HIFs can be targeted as the potential candidate to reeducate TAM. This is because HIFs are overexpressed by TAM that resides in the hypoxic TME. In addition, HIFs are able to induce angiogenic factor production such as VEGF and IL-8 in HIF-1 knockout mouse model [81]. Another study conducted by Doedens et al. [82] has showed an interesting result in which HIF-1*α* (a subtype of HIF) is able to reduce the growth of tumour in mice.

An accumulating evidence has shown that microRNA-155 (miRNA-155) is an interesting target candidate to educate macrophages in TAM. Masaki et al. [83] claimed that miRNA-155 is essential in regulating myelopoiesis and erythropoiesis from CD34^+^ hematopoietic stem progenitor cells. miRNA-155 will be rapidly expressed as a response to infection or injury. However, miRNA-155 is able to be induced by alarmins [84], pathogen-associated molecular patterns (PAMPs) and damage-associated molecular patterns (DAMPs) [85], and inflammatory stimuli such as TNF and IL-1*β* [86]. A recent study conducted by Liu et al. [87] managed to prove the importance of miRNA-155 in regulating the development of colon cancer in CRC patients. In the study, they investigated the interaction of miRNA-155 with collagen triple helix repeat containing 1 (CTHRC1) in the pathogenesis of CRC. From their study, they found out that the expression of miRNA-155 is inversely proportional with the expression of CTHRC1. In addition, the result showed that the overexpression of miRNA-155 managed to suppress the cell proliferation as well as promote the apoptosis of HT-29 cells by silencing the CTHRC1 expression.

Poh et al. [88] reported that hematopoietic cell kinase (HCK) is able to trigger hematological malignancies by acting as tumour cell-intrinsic oncogene in C57BL/6 cancer mice model. HCK is a member of SRC family nonreceptor tyrosine kinase (SFKs) that mainly express in B lymphocytes [88, 89]. The study also showed that high HCK level will cause the lower survival rate of CRC patient due to the accumulation of M2 in TAM. Thus, the inhibition of HCK activity may suppress or disrupt the activation of M2 macrophage and eventually will regress the proliferation of CRC [88].

## 6. Conclusion

CRC is a progressive cancer which encompasses complex processes. Multiple cellular pathways are involved starting from the initial process of tumour transformation to metastasis. The cellular pathways play an important role in determining the TAM activity. An increasing number of studies have indicated that TAM can be a suitable candidate to be targeted for anticancer therapy [90]. Several approaches have now been taken into consideration in designing an experiment [71]. The approaches taken are aimed at suppressing the protumoural activity of macrophage in cancer development which can potentially be the cure for cancer [91]. One of the approaches is to reeducate the TAM from having protumoural property to become antitumoural activity.

Various candidates have been identified to be suitable in reeducating TAM. However, the pathways such as VEGF and NF-*κ*B that regulate TAM activity can be tricky. Thus, it is essential to comprehensively understand the pathways involved in regulating TAM activity in order to tackle such barrier. We hope that some of the candidates as mentioned in the previous section can be utilised either as single use or as combination to improve the efficiency of current conventional therapies. Moreover, it would be better if the specificity of the candidates can be improved so that the candidates could be specifically delivered to the TAM.

## Figures and Tables

**Table 1:** Estimated new cases of CRC in the United States by sex in 2017 (adapted and modified from Siegel et al. [4]).

Body part	Estimated new cases	
Digestive system	Male	Female
Colon	47,700	47,820
Rectum	23,720	16,190

**Table 2:** Malaysia CRC incidence rate per 100,000 by sex and ethnicity from 2008 to 2013 (adapted and modified from Abu Hassan et al. [8]).

Characteristic	CRC incidence (per 100,000)		
Ethnicity	Overall	Male	Female
Malay	18.95	21.79	16.09
Chinese	27.35	30.77	23.22
Indian	17.55	21.43	13.71
